# A Trypsin Inhibitor from *Tecoma stans* Leaves Inhibits Growth and Promotes ATP Depletion and Lipid Peroxidation in *Candida albicans* and *Candida krusei*

**DOI:** 10.3389/fmicb.2016.00611

**Published:** 2016-04-27

**Authors:** Leydianne L. S. Patriota, Thamara F. Procópio, Maria F. D. de Souza, Ana Patrícia S. de Oliveira, Lidiane V. N. Carvalho, Maira G. R. Pitta, Moacyr J. B. M. Rego, Patrícia M. G. Paiva, Emmanuel V. Pontual, Thiago H. Napoleão

**Affiliations:** ^1^Departamento de Bioquímica, Universidade Federal de PernambucoRecife, Brazil; ^2^Laboratório de Imunomodulação e Novas Abordagens Terapêuticas, Núcleo de Pesquisa em Inovação Terapêutica, Universidade Federal de PernambucoRecife, Brazil; ^3^Departamento de Morfologia e Fisiologia Animal, Universidade Federal Rural de PernambucoRecife, Brazil

**Keywords:** protease inhibitor, antifungal activity, *Candida*, oxidative stress, anionic protein

## Abstract

*Tecoma stans* (yellow elder) has shown medicinal properties and antimicrobial activity. Previous reports on antifungal activity of *T. stans* preparations and presence of trypsin inhibitor activity from *T. stans* leaves stimulated the investigation reported here. In this work, we proceeded to the purification and characterization of a trypsin inhibitor (TesTI), which was investigated for anti-*Candida* activity. Finally, in order to determine the potential of TesTI as a new natural chemotherapeutic product, its cytotoxicity to human peripheral blood mononuclear cells (PBMCs) was evaluated. TesTI was isolated from saline extract by ammonium sulfate fractionation followed by ion exchange and gel filtration chromatographies. Antifungal activity was evaluated by determining the minimal inhibitory (MIC) and fungicide (MFC) concentrations using fungal cultures containing only yeast form or both yeast and hyphal forms. *Candida* cells treated with TesTI were evaluated for intracellular ATP levels and lipid peroxidation. Cytotoxicity of TesTI to PBMCs was evaluated by MTT assay. TesTI (39.8 kDa, pI 3.41, *K*_i_ 43 nM) inhibited similarly the growth of both *C. albicans* and *C. krusei* culture types at MIC of 100 μg/mL. The MFCs were 200 μg/mL for *C. albicans* and *C. krusei*. Time-response curves revealed that TesTI (at MIC) was more effective at inhibiting the replication of *C. albicans* cells. At MIC, TesTI promoted reduction of ATP levels and lipid peroxidation in the *Candida* cells, being not cytotoxic to PBMCs. In conclusion, TesTI is an antifungal agent against *C. albicans* and *C. krusei*, without toxicity to human cells.

## Introduction

The chemical protection of plants comprises several compounds, including different classes of defense proteins (Carlini and Grossi-de-Sá, 2002; Dang and Van Damme, 2015). The insecticidal and antimicrobial effects of these proteins against pathogens and predators have drawn the attention of researchers for their potential biotechnological applications (Paiva et al., 2013a,b).

Protease inhibitors are compounds that block or reduce the activity of proteolytic enzymes, acting in regulation of endogenous proteolytic activity. The participation of proteinaceous protease inhibitors in the defense mechanisms of plants and animals has been reported (Habib and Fazili, 2007; Zhu-Salzman and Zeng, 2015) and, in this sense, these molecules have been evaluated for insecticidal and antimicrobial properties (Fear et al., 2007; Kim et al., 2009; Soares et al., 2011; Paiva et al., 2013a,b). Their antimicrobial activity can be attributed to the ability to inhibit extracellular and/or intracellular proteases (Paiva et al., 2013a). In addition, protease inhibitors can also promote damage of cell membrane/wall, alteration of cell permeability, inhibition of transcription and/or translation, and induction of reactive oxygen species (ROS) production (Paiva et al., 2013a; Macedo et al., 2016; Majchrzak-Gorecka et al., 2016).

*Tecoma stans* (L.) Kunth (Bignoniaceae), also known as yellow elder, is a plant native to the North and Central Americas, which was introduced in Brazil for ornamental purposes and became abundant due to the ease of seed dispersion, rapid growth, and relative absence of natural enemies (Passini and Kranz, 1997; Silva et al., 2008). *T. stans* leaves are often used in the treatment of diabetes and have been used in the synthesis of nanoparticles (Arunkumar et al., 2013; Cruz and Andrade-Cetto, 2015). Extracts and compounds (tannins, flavonoids, alkaloids, quinones, and saponins) from this plant have shown antibacterial, antifungal, antitumoral, and hypoglycemic activities (Binutu and Lajubutu, 1994; Aguilar-Santamaría et al., 2009; Ramesh et al., 2009; Kameshwaran et al., 2012). For example, an ethanolic extract from *T. stans* leaves showed inhibitory and fungicidal effect on *Candida tropicalis* and *C. albicans* (Al-Judaibi and Al-Yousef, 2014). Additionally, *T. stans* is considered a plant resistant to pests and pathogens (Gilman and Watson, 1994) and thus is attractive for research purposes involving defense proteins.

Our laboratory works on the detection and isolation of bioactive proteins from plants, mainly focused on lectins and protease inhibitors. Since no information was found in the literature about the presence of these proteins in *T. stans*, we conducted a preliminary study that indicated that leaves of this plant contain trypsin inhibitor activity while lectins were not detected (Patriota et al., 2014). In this sense, the investigation reported here was stimulated by the presence of trypsin inhibitor in *T. stans* leaves together with the previous reports on antifungal activity of *T. stans* preparations. In the present work, we report the purification and characterization of a trypsin inhibitor from *T. stans* leaves (TesTI) and its investigation for antifungal activity against *Candida* species.

The yeasts from the *Candida* genus (Class Saccharomycetes, Order Saccharomycetales) are opportunistic and pathogenic fungi that cause cutaneous and mucosal infections; they also cause systemic infections, mainly in immunocompromised patients (Lima et al., 2008; Papon et al., 2013). There is an increase in reports on the emergence of *Candida* resistant strains, which may emerge even still during the course of medical treatment (Arendrup, 2013). In this sense, the search for natural compounds with anti-*Candida* activity has been stimulated (Rybalchenko et al., 2010; Kim et al., 2012; Zhang et al., 2014; Yessoufou et al., 2015). Antimicrobial peptides from animals and plants have shown anti-*Candida* activity (Aerts et al., 2011; Cho and Lee, 2011) and the inhibition of proteases secreted by *Candida* species is considered as an interesting antifungal strategy (Stewart and Abad-Zapatero, 2001). Recently, Macedo et al. (2016) reported that a trypsin inhibitor from *Inga laurina* seeds was able to inhibit the growth of *C. tropicalis* and *C. buinensis*.

Finally, since compounds with high toxicity against human cells cannot be considered potential chemotherapeutic products, we determined the cytotoxicity of TesTI to human peripheral blood mononuclear cells (PBMCs).

## Materials and Methods

### Protein Extraction

Leaves were collected in the campus of the *Universidade Federal de Pernambuco* (UFPE), with authorization (number 36301) of the *Instituto Chico Mendes de Conservação da Biodiversidade* (ICMBio). The leaves were dried at 28°C for 7 days and powdered using a blender. The leaf powder was then homogenized (16 h, 28°C) at 10% (w/v) in 0.15 M NaCl using a magnetic stirrer. The homogenate was filtered using a filter paper and the filtrate was used as the extract. Protein concentration was measured according to the method described by Lowry et al. (1951) using a standard curve of bovine serum albumin (31.25–500 μg/mL).

### Trypsin Inhibitor Activity

Assays for determination of trypsin inhibitor activity were performed in 96-well microplates according to the protocol described by Pontual et al. (2014). Bovine trypsin (5 μL; 0.1 mg/mL) in 0.1 M Tris-HCl pH 8.0 (Tris buffer) was incubated with 30 μL of the leaf extract for 15 min at 28°C. Then, 5 μL of 8 mM *N*-benzoyl-DL-arginyl-*p*-nitroanilide (BApNA) was added. The volume of each well was adjusted to 200 μL with Tris buffer. Trypsin activity in the absence of extract was considered as 100% substrate hydrolysis. Blanks were performed submitting the extract to the same reaction steps in the absence of substrate and enzyme. Also, blanks of the substrate in the absence of enzyme and sample were prepared. One unit of trypsin activity was defined as the amount of enzyme that hydrolyzes 1 μmol of BApNA per minute. Each milli-unit (mU) of trypsin activity inhibited by the presence of extract corresponded to one unit of trypsin inhibitor activity. Specific trypsin inhibitor activity was calculated as the ratio of trypsin inhibitor activity (U) to the amount of protein (mg) in the sample.

### Isolation and Characterization of *T. stans* Trypsin Inhibitor (TesTI)

The leaf extract was treated with ammonium sulfate at 80% saturation (Green and Hughes, 1955) using magnetic stirring for 4 h at 28°C. After centrifugation (3,000 ×*g*; 15 min) the precipitate and supernatant fractions were collected and dialyzed against distilled water (2 h) and Tris buffer (2 h). The precipitate fraction (2 mL; 3.6 mg of protein) was loaded onto a DEAE-cellulose (Sigma–Aldrich, USA) column (7.5 cm × 1.5 cm) equilibrated with Tris buffer (20 ml/h). After washing, the adsorbed proteins were eluted with 1.0 M NaCl prepared in the equilibration buffer. Fractions of 2.0 mL were collected and dialyzed against 0.15 M NaCl (1 L; 4 h).

The PII pool (eluted from DEAE-cellulose chromatography) was loaded (2 mL; 0.274 mg protein) onto a gel filtration HiPrep 16/60 Sephacryl S-100 HR column coupled to the ÄKTAprime plus system (GE Healthcare Life Sciences, Sweden). Chromatography was performed using 0.15 M NaCl at a flow rate of 0.5 mL/min. The fractions (2 mL) collected were pooled into three groups (PIIa, PIIb, and PIIc) based on the absorbance at 280 nm. The range of molecular mass of the components in each pool was estimated by comparison with the migration times of protein molecular weight markers (14.4–97 kDa) chromatographed under the same conditions.

PIIa was then dialyzed against Tris buffer (1 L; 2 h) and loaded (2 mL; 0.134 mg protein) onto a DEAE FF 16/10 column (GE Healthcare Life Sciences, Sweden) coupled to the ÄKTAprime system and previously equilibrated with Tris buffer (5.0 mL/min). The adsorbed proteins were eluted using a gradient of 1.0 M NaCl (0–100%) in Tris buffer. TesTI was enriched in the pool of fractions eluted with 50–64% NaCl. The purification was monitored by measuring the protein concentration and trypsin inhibitor activity of all fractions.

TesTI (150 μg) was resuspended in rehydration buffer [8.0 M urea, 2% (w/v) CHAPS, 1% (v/v) IPG buffer pH 3–10 and 0.002% (w/v) bromophenol blue] and absorbed by the strip (7 cm; linear pH gradient 3–10) passively for 16 h at 25°C. The rehydrated strip was submitted to isoelectric focusing using the Ettan IPGPhor III system (GE Healthcare Life Sciences, Sweden) according to the manufacturer’s instructions. The strip was then washed three times with an equilibrating solution [50 mM Tris-HCl pH 8.8, 6.0 M urea, 30% (v/v) glycerol, 2% (w/v) SDS and 0.002% (w/v) bromophenol blue] and transferred to the top of a 12% (w/v) polyacrylamide gel containing SDS (Laemmli, 1970). Molecular mass standards (12–225 kDa, GE Healthcare Life Sciences) were also subject to electrophoresis. The polypeptides were stained with 0.02% (w/v) Coomassie Blue G. The gel was analyzed using the Image Master (GE Healthcare) software.

The Dixon plot analysis was used to determine the inhibitory constant (*K*_i_) of TesTI for bovine trypsin (Segel, 1975). Trypsin inhibition assay was carried out using two BApNA concentrations (4 and 8 mM). TesTI solutions prepared at concentrations ranging from 0.56 to 1.88 μM.

### Antifungal Assay

Stored cultures of *C. albicans* (URM 5901), *C. tropicalis* (URM 6551), and *C. krusei* (URM 6391) were obtained from the Culture Collections of the University Recife Mycologia, *Departamento de Micologia* (UFPE). The fungal cultures were reactivated in Sabouraud-Dextrose for 48 h at 30°C (to obtain a yeast culture) or 37°C (to obtain a culture containing yeast and hyphal forms) on a shaker. The culture concentration was adjusted to 3 × 10^6^ colony-forming units (CFU) per mL for use in the assays.

Minimal inhibitory concentrations (MIC) of leaf extract, precipitate fraction and TesTI were determined using 96-well microtiter plates and both culture types. Each row of the plate corresponded to an antifungal assay. First, 100 μL of Sabouraud-Dextrose was added to each well, followed by the sample (100 μL) which was added to the third well and serially diluted (twofold dilution) until the last well of the row. All the wells except the first were inoculated with 20 μL of the fungal culture. The first well corresponded to the negative control (contained only culture medium) and the second well corresponded to the 100% growth control (containing a 1:1 dilution of culture medium in water). The plate was read at time zero and after 24 h using a microplate reader at 600 nm (OD_600_). In this time interval, the plate was incubated at 30°C (assays using yeast cultures) or 37°C (assays using yeast/hyphae cultures). Each assay was performed in triplicate and two independent experiments were performed. The MIC was defined as the lowest sample concentration that could reduce the OD_600_ by 50% or higher than that of 100% growth control (Amsterdam, 1996). The minimal fungicide concentrations (MFCs) were determined using the MIC assays as reference. Aliquots of the wells corresponding to concentrations ≥MIC and 100% growth control were transferred to petri plates containing Sabouraud-Dextrose-Agar and incubated for 24 h at 30 or 37°C. MFC was defined as the concentration that reduced the number of CFU by least 99.9% in comparison to the initial inoculum.

### Growth Curves of *C. albicans* and *C. krusei*

Growth curves for *C. albicans* and *C. krusei* were established in the absence and presence of TesTI. The assay was performed in 96-well microplates using three wells of each row: the first well corresponded to the negative control and the second well corresponded to the 100% growth control, both prepared as described above; the third well corresponded to the treatment with TesTI at MIC. The microplate was incubated at 37°C and the optical density at 600 nm was determined every hour until 24 h. Two experiments were performed in duplicate.

### ATP Quantification Assay

The quantification of intracellular ATP was performed using the CellTiter-Glo Luminescent Cell Viability Assay kit (Promega Corporation, USA), which is based on the reaction between the luciferase from *Photuris pennsylvanica* (LucPpe2) and ATP molecules generating a luminescent signal. *C. albicans* and *C. krusei* cells (10^6^ CFU) were treated for 24 h at 37°C with TesTI at MFC, MIC, aaa MIC, and bbb MIC. A 100% growth control (non-treated cells) was also performed as described in Section “Antifungal Assay.” After this period, the microplate was maintained for 30 min at 25°C and then the cells were transferred to wells of an opaque-walled 96-well plate (100 μL per well). Next, 100 μL of Cell Titer-Glo^®^ reagent was added to each well and the contents were mixed for 2 min on an orbital shaker to induce cell lysis. The plate was incubated for 10 min at 25°C and then the luminescence was recorded. Three independent experiments were performed in triplicate. The results were expressed as relative ATP levels, which were calculated by the ratio between the level in the test assay and that in the control assay.

### Lipid Peroxidation Assay

The assay was performed using the Lipid Peroxidation (MDA) Assay Kit (MAK085) from Sigma–Aldrich (USA). *C. albicans* and *C. krusei* cultures (10^6^ CFU) were incubated with TesTI at the MIC in 0.15 M NaCl for 24 h at 37°C. The cells were disrupted using a lysis buffer (300 μL) containing 3 μL of butylated hydroxytoluene (100×) and the samples were centrifuged at 13,000 ×*g* for 10 min. The supernatants (200 μL) were collected in microcentrifuge tubes and 600 μL of thiobarbituric acid (TBA) was added to each tube. The assays were incubated for 60 min at 95°C and then in an ice bath for 10 min. The absorbance was read at 532 nm using a spectrophotometer. Reactions performed in absence of cell lysates were used as blanks. The concentration of malondialdehyde (end product of lipid peroxidation) was determined using a standard curve (0–20 nmol). Three independent experiments were performed in triplicate.

### Cytotoxicity Assay

Peripheral blood mononuclear cells were obtained from heparinized blood from healthy and non-smoking donors (*n* = 3) who had not taken any drugs for at least 15 days before the sample collection. The PBMCs were isolated using a standard method of density-gradient centrifugation over Ficoll-Hypaque (GE Healthcare Life Sciences, Sweden). Cells were counted in a Neubauer chamber and viability was determined by the trypan blue exclusion method. Cells were only used in the assays when viability was higher than 98%. All donors signed an informed consent form and the study was approved by the Human Research Ethics Committee of the *Universidade Federal de Pernambuco* (CEP/CCS/UFPE N° 145/09).

In each assay, the PBMCs (1 × 10^6^ cells/mL) were plated in 96-well microtiter plates and, after 24 h, TesTI (in 0.15 M NaCl) was added to different wells in order to obtain the concentrations 10 and 100 μg/mL. After incubation (48 h at 37°C), 100 μL of a 3-(4,5-dimethylthiazol-2-yl)-2,5-diphenyl tetrazolium bromide (MTT) solution (5.0 mg/mL) was added to each well, and the cell viability was determined by assessing the ability of the viable cells to reduce the yellow tetrazolium to a blue formazan product. After 3 h, the formazan was dissolved in 20% (w/v) sodium dodecyl sulfate (SDS), and absorbance at 570 nm was measured using a multi-plate reader (ELX 800, Biotek, USA). The effect of TesTI was quantified as percentage of the absorbance of the reduced dye in control (cells treated only with 0.15 M NaCl). The assays were performed in triplicate in two independent experiments.

### Statistical Analysis

Means and standard deviations (SD) were calculated and data were expressed as a mean of replicates ± SD. Significance between groups was determined by Student’s *t*-test or Tukey’s test at significance level of *p* < 0.05.

## Results

The extract from *T. stans* leaves showed protein concentration of 23.52 mg/mL and trypsin inhibitor activity of 93.0 U/mg. In order to promote a partial purification of the proteins, the extract was submitted to treatment with ammonium sulfate. The precipitate fraction obtained showed higher specific trypsin inhibitor activity (1,540 U/mg). The supernatant fraction could also reduce the activity of trypsin; however, its specific activity (600 U/mg) was lower than that of the precipitate fraction. Then, the precipitate fraction was chosen to continue the purification process, being the starting point for the chromatographic steps.

**FIGURE 1:**
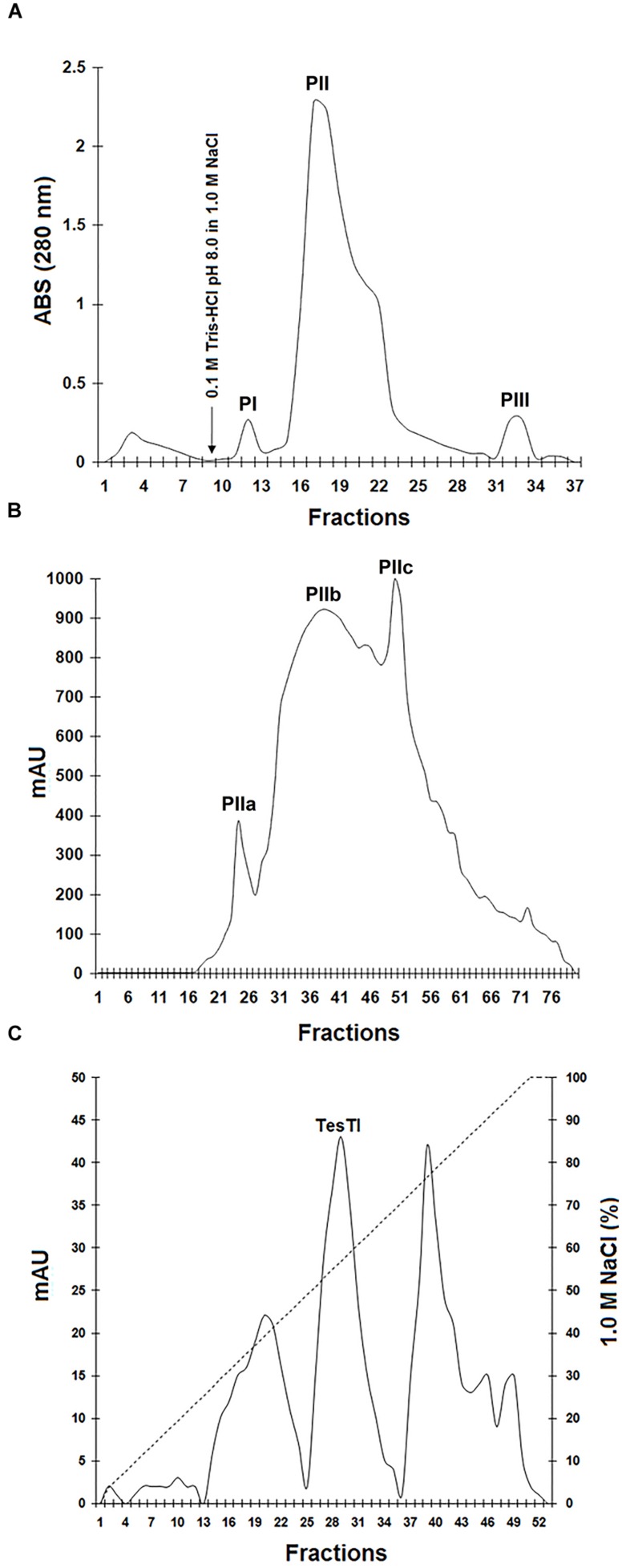
**Purification of trypsin inhibitor from *Tecoma stans* leaves (TesTI). (A)** Chromatography of precipitate fraction (obtained after treatment of leaf extract with ammonium sulfate) on a DEAE-cellulose column. Washing was performed using 0.1 M Tris-HCl pH 8.0. The arrow indicates the time of addition of the eluent (1.0 M NaCl). PI: fractions 11–13; PII: 15–27; PIII: 32–33. **(B)** Gel filtration chromatography of PII (from DEAE-cellulose column) on HiPrep 16/60 Sephacryl S-100HR column coupled to the ÄKTAprime plus chromatography system. Three pools were separated: PIIa (fractions 22–27), PIIb (28–47) and PIIc (48–58). **(C)** Chromatography of PIIa in the ion exchange DEAE FF 16/10 column coupled to the ÄKTAprime plus system and equilibrated with 0.1 M Tris-HCl pH 8.0. The elution step was performed using a 1.0 M NaCl gradient (0–100%). TesTI corresponded to the pool of fractions 26–33, eluted with 50–64% of 1.0 M NaCl. Chromatographies were monitored by measuring the absorbance at 280 nm.

Initially, the precipitate fraction was submitted to chromatography on DEAE-Cellulose column. As can be seen in **Figure 1A**, three peaks of adsorbed proteins were detected. The pool PII showed the highest specific trypsin inhibitor activity (1,833 U/mg). Nevertheless, it was still quite pigmented and showed several polypeptide bands with low resolution in polyacrylamide gel electrophoresis (data not shown), indicating the necessity of another chromatography step. This second step consisted in a gel filtration chromatography, which separated PII in three peaks (**Figure 1B**). The pool PIIa, corresponding to molecules with mass ranging from 23.2 to 47.5 kDa, showed no color and inhibited trypsin at 8421 U/mg. PIIb (molecules with mass ranging from 4.7 to 23.1 kDa) was strongly colored and also showed trypsin inhibitor activity (4794 U/mg). PIIc (molecules with mass < 4 kDa) was highly pigmented and did not inhibit trypsin.

**FIGURE 2:**
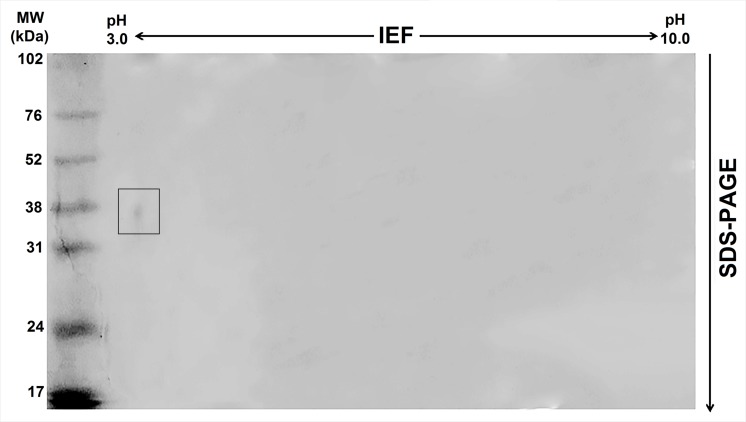
**Two-dimensional electrophoresis of the trypsin inhibitor from leaves of *T. stans* (TesTI).** A single spot, corresponding to a relative molecular mass of 39.8 kDa and a pI of 3.41, was detected. The gel was stained with Coomassie Brilliant Blue.

Finally, a third chromatography step was performed by submitting the pool PIIa to an ion exchange DEAE FF 16/10 column. In the elution step, performed using a 1.0 M NaCl gradient (0–100%), three peaks were recovered (**Figure 1C**). Only the peak corresponding to fractions eluted with 50–64% of 1.0 M NaCl inhibited trypsin, and this was named TesTI (*T. stans* trypsin inhibitor). **Table 1** summarizes the process of TesTI purification. Two-dimensional electrophoresis of TesTI (**Figure 2**) yielded a single spot, with molecular weight of 39.8 kDa and pI of 3.41. TesTI showed a *K*_i_ of 43 nM toward bovine trypsin. Once confirmed the homogeneity of TesTI, we started the evaluation of antifungal activity.

**Table 1:** Summary of purification of the trypsin inhibitor from *Tecoma stans* leaves (TesTI).

Sample	Protein concentration (mg/mL)	Specific inhibitor activity (U/mg)	Purification (fold)
Leaf extract	23.52	93	1.0
Precipitate fraction	1.8	1540	16.6
PII	0.137	1833	19.7
PIIa	0.067	8421	90.5
TesTI	0.046	22,400	240.8

*Specific trypsin inhibitor activity corresponds to the ratio of trypsin inhibitor activity (U) and the amount of protein (mg) in the sample in the assay. Precipitate fraction obtained after treatment of leaf extract with ammonium sulfate (80% saturation). PII: main protein peak eluted from the chromatography of precipitate fraction on the ion exchange column DEAE-cellulose. PIIa: main protein peak obtained from chromatography of PII on HiPrep 16/60 Sephacryl S-100 HR column. TesTI was recovered after chromatography of PIIa on ion exchange column DEAE FF 16/10. Purification factor corresponded to the ratio of the specific trypsin inhibitor activity of each sample to that of the leaf extract.*

Antifungal activity was evaluated for the leaf extract, precipitate fraction, and TesTI; the results are shown in **Table 2**. Similar results were obtained in assays performed with cultures containing only yeast form (30°C) or both yeast and hyphal forms (37°C). The leaf extract did not show antifungal activity, while the fraction inhibited the growth of all species. TesTI was more effective than the fraction in inhibiting the growth of *C. albicans* and *C. krusei* but was not active against *C. tropicalis*. No fungicide activity was detected in the fraction, while TesTI was cytotoxic to *C. albicans* and *C. krusei* (**Table 2**).

**Table 2:** Antifungal activity of leaf extract, precipitate fraction, and TesTI against *Candida* species.

Fungi	Extract		Precipitate fraction		TesTI	
	Yeast culture	Yeast/hyphae culture	Yeast culture	Yeast/hyphae culture	Yeast culture	Yeast/hyphae culture
***Candida albicans***						
MIC (μg/mL)	ND	ND	5,200	2,600	100	100
MFC (μg/mL)	–	–	>5,200	>5,200	200	200
***Candida krusei***						
MIC (μg/mL)	ND	ND	2,600	2,600	100	100
MFC (μg/mL)	–	–	>5,200	>5,200	200	200
***Candida tropicalis***						
MIC (μg/mL)	ND	ND	2,600	2,600	ND	ND
MFC (μg/mL)	–	–	>5,200	>5,200	–	–

*Antifungal assays were performed at 30°C (using yeast cultures) or 37°C (using yeast/hyphae cultures). MIC (minimal inhibitory concentration) and MFC (minimal fungicide concentration) are expressed as protein concentration. Precipitate fraction obtained after treatment of leaf extract with ammonium sulfate (80% saturation). ND, not detected. –, not evaluated, since inhibitory activity was not observed.*

**FIGURE 3:**
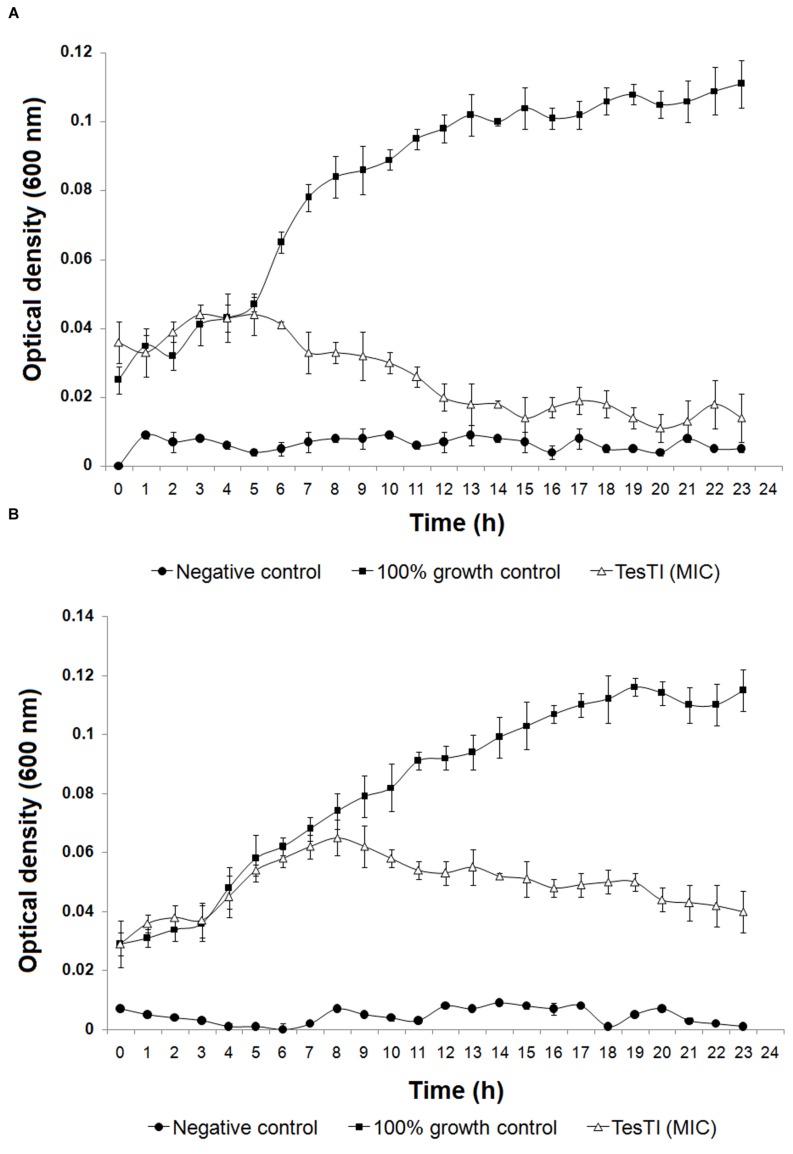
**Growth curves of *Candida albicans* (A) and *Candida krusei***(B)** in absence and presence of TesTI at MIC.** The absorbance (600 nm) was determined every hour during 24 h. Negative control contained only culture medium and did not receive microbial culture. The 100% growth control contained a 1:1 dilution of culture medium in water. Data are expressed as the mean ± standard deviation (SD).

**FIGURE 4:**
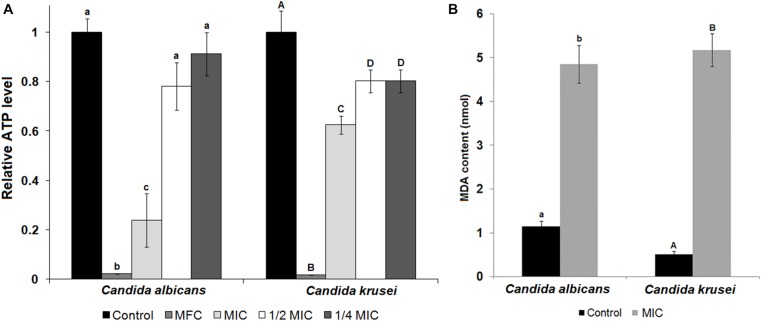
**ATP and lipid peroxidation levels of untreated and TesTI-treated *C. albicans* and *C. krusei* cells. (A)** ATP levels in TesTI-treated cells at MFC, MIC, aaa MIC, and bbb MIC. The relative ATP levels were calculated by the ratio between the value in the test assay and that in the control assay (which was considered 1.0). **(B)** Malondialdehyde (MDA) levels from cells of control and TesTI-treated at MIC. Different letters indicate significant differences between treatments. Lowercase letters were used for comparison of *C. albicans* results and uppercase letters for *C. tropicalis.*

The species that were sensitive to TesTI had their growth curves at 37°C established when incubated or not with the inhibitor at the MIC. It is possible to note that the growth of *C. albicans* was significantly (*p* < 0.05) different in regard to 100% growth control from the sixth hour (**Figure 3A**) while that of *C. krusei* was significantly (*p* < 0.05) reduced from the ninth hour (**Figure 3B**). **Figure 4A** shows that ATP levels were not significantly different (*p* > 0.05) in *C. albicans* cells and control when incubated with TesTI at aaa MIC and bbb MIC; however, the levels were significantly lower (*p* < 0.05) in cells treated with TesTI at MIC. For *C. krusei*, the ATP levels were significantly (*p* < 0.05) lower when treated at MIC, aaa MIC, and bbb MIC. As expected, the ATP levels in *C. albicans* and *C. krusei* incubated at MFC were negligible. At MIC, TesTI induced lipid peroxidation in *C. albicans* and *C. krusei* since malondialdehyde content for treated cells was higher than that of control cells (**Figure 4B**).

**FIGURE 5:**
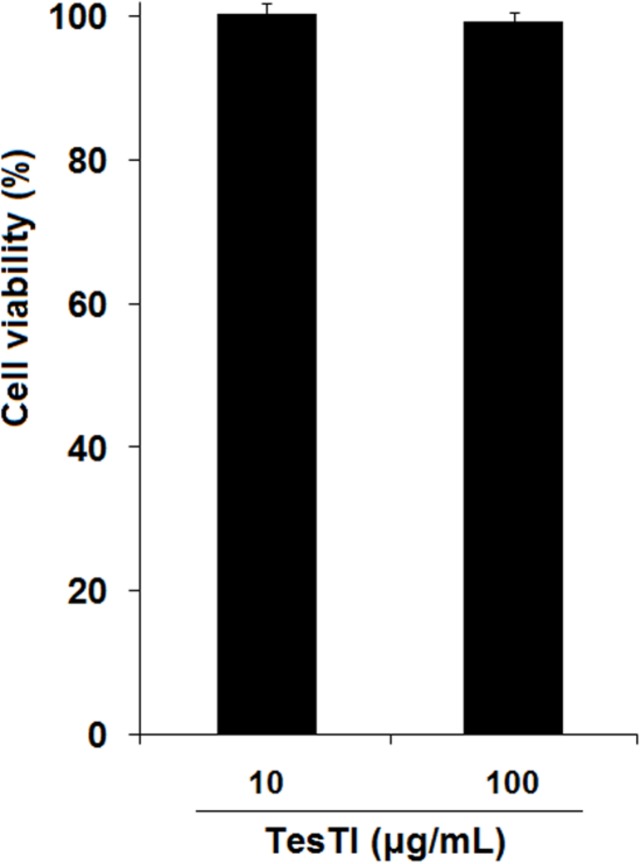
**Viability of human peripheral blood mononuclear cells (PBMCs) incubated with TesTI (10 and 100 μg/mL).** Results are expressed as percentage of viability in comparison with control using 0.15 M NaCl (100% viability).

Based on the MIC values found in antifungal assay, we determined the toxicity of TesTI to non-target cells. In the cytotoxicity assays, the vehicle (0.15 M NaCl) did not alter the viability of PBMCs and thus the results were used as the 100% growth control. TesTI (10 and 100 μg/mL) was not cytotoxic agent to normal PBMCs since there was no reduction in cell viability in comparison with control (**Figure 5**).

## Discussion

The previous reports on antimicrobial properties of protease inhibitors, the presence of trypsin inhibitor (a class of defense proteins) in *T. stans* leaves and the emergence of *Candida* strains resistant to currently available chemotherapeutic agents prompted us to conduct this study. Given that the leaves of *T. stans* contain trypsin inhibitor, we undertook efforts in order to isolate this protein and to evaluate whether it would have potential for application as an antifungal agent. If this antifungal potential was proven, our planning would follow with the evaluation of action mechanisms and cytotoxicity to human cells.

The isolation of TesTI included an ammonium sulfate fractionation and three chromatographic steps. The higher specific trypsin inhibitor activity of precipitate fraction in comparison with leaf extract and supernatant fraction indicates that protein precipitation resulted in partial purification of trypsin inhibitor molecules.

The results from chromatography of precipitate fraction on DEAE-Cellulose column indicated that most of the proteins in the fraction were anionic in nature, since they adsorbed to the cationic matrix. The high pigmentation and the detection of several polypeptide bands in electrophoresis lead us to perform the other chromatographic steps described above. Since the pool PIIa, from gel filtration chromatography, was colorless and showed the highest specific activity, it was chosen for the chromatography on DEAE FF 16/10 column, which finally yielded a homogeneous protein preparation containing TesTI. The isoelectric focusing confirmed TesTI as an anionic protein as indicated since the first chromatography step.

Following the successful isolation of TesTI, we evaluated its antifungal potential. The strains used were clinical isolates obtained from ungula samples (*C. albicans*), oral cavity tumor (*C. tropicalis*), and blood of a candidemia patient in ICU (*C. krusei*). The results for leaf extract and precipitate fraction show that salt fractionation was effective in concentrating antifungal agents that were diluted in the extract. The results obtained with TesTI reveal an interesting specificity of the inhibitor, particularly with respect to the different effects on *C. albicans* and *C. tropicalis*, which are closely related taxonomically (Chai et al., 2010). The fact that TesTI was not active against *C. tropicalis* indicates that the *T. stans* leaves contain other antifungal agents than the trypsin inhibitor isolated in this work.

The other assays were then performed using the yeast/hyphae cultures since the results from assays performed at 30 and 37°C were similar and the hyphal form is closer to the situation of the pathogen in human body. The time-response curve for *C. albicans* indicates that TesTI not only disrupts the replication of the cells at MIC but also promotes cell death since the optical density at 24 h was lower than that in the start of the experiment. In addition, the curves indicate that TesTI was more effective on *C. albicans* than *C. krusei*.

Incubation of fungal cells with TesTI at MIC and MFC resulted in decreasing of the ATP content for both *Candida* strains, which may be due to a lower number of viable cells. In the treatments at the MIC, the ATP level was much lower for *C. albicans* than that for *C. krusei*, which is in accordance with the result of the growth curves, which also indicated a lower viability of *C. albicans*. With respect to treatments with concentrations below, it was detected ATP levels significantly (*p* < 0.05) lower than in the control only for *C. krusei*. This may be linked to an initial disruption of energy metabolism, since ATP levels decreased even at sub-inhibitory concentrations. ATP depletion is usually associated with damage to mitochondria caused by oxidative stress (Al-Dalaen and Al-Qtaitat, 2014), initiated by alterations in the redox balance in biological systems due to predominance of pro-oxidant species (usually ROS). Most of the cellular components (proteins, carbohydrates, lipids, and DNA) are potential targets of ROS, which cause loss of function and induce cell death. Since our assays revealed a decrease in ATP levels at inhibitory and sub-inhibitory concentrations of TesTI, we investigated whether the inhibitor induced oxidative stress in *Candida* cells by determining the levels of lipid peroxidation. Indeed, lipid peroxidation was detected.

The lipid peroxidation may occur through direct action of the ROS or by a secondary mechanism involving the products of lipid peroxidation. At the membrane, lipid peroxidation propagates the formation of new peroxyl radicals, which destabilize the membrane and affect the function of receptors, signal transducers, transport proteins and enzymes (Halliwell and Gutteridge, 2007). Macedo et al. (2016) showed that the trypsin inhibitor from *I. laurina* seeds induced the production of ROS in *C. tropicalis* and *C. buinensis* cells after incubation for 24 h and the authors suggested that the inhibitor could have a mitochondrial target. Our study with TesTI is another report on the induction of oxidative stress by a proteinaceous trypsin inhibitor.

Other peptides have induced oxidative stress in *Candida* cells. The antimicrobial peptide pleurocidin (from *Pleuronectes americanus* skin mucous) induced the production of intracellular ROS in *C. albicans*, which resulted in DNA fragmentation and mitochondrial membrane depolarization (Cho and Lee, 2011). A peptide from *Heuchera sanguinea* seeds exerted antifungal activity by interfering with mitochondrial functionality, causing accumulation of ROS in cells of *Saccharomyces cerevisiae* and *C. albicans* (Aerts et al., 2011).

Since compounds with high toxicity against human cells would not be viable for use as antifungal agents, we evaluated the toxicity of TesTI to human PBMCs. The inhibitor did not show any toxicity at concentrations of 10 and 100 μg/mL, indicating that it can be considered as a potential drug to control *Candida* infections.

## Conclusion

The TesTI is an anionic protein with no toxicity to human PBMCs and that promotes oxidative stress in *C. albicans* and *C. krusei*, which probably impairs energy metabolism. A decrease in the availability of ATP for use in metabolic reactions probably affected morphogenesis and division of *Candida* cells, resulting in growth inhibition and cell death.

## Author Contributions

Conceived and designed the experiments: LP, TP, AO, MP, MR, EP, and TN. Performed the experiments: LP, TP, MS, AO, LC, EP, and TN. Analyzed the data: LP, TP, MS, AO, LC, MP, MR, PP, EP, and TN. Contributed reagents/materials/analysis tools: MP, MR, PP, and TN. Wrote the paper: LP, TP, EP, and TN.

## Conflict of Interest Statement

The authors declare that the research was conducted in the absence of any commercial or financial relationships that could be construed as a potential conflict of interest.
